# Current Approach in the Diagnosis and Management of Uveitic Glaucoma

**DOI:** 10.1155/2015/742792

**Published:** 2015-10-19

**Authors:** Francisco J. Muñoz-Negrete, Javier Moreno-Montañés, Paula Hernández-Martínez, Gema Rebolleda

**Affiliations:** ^1^Ophthalmology Service, Hospital Universitario Ramón y Cajal, Instituto Ramón y Cajal de Investigaciones Sanitarias (IRYCIS), Madrid, Spain; ^2^Universidad de Alcalá, Alcalá de Henares, Spain; ^3^Ocular Pathology National Net (OFTARED) of the Institute of Health Carlos III, Madrid, Spain; ^4^Department of Ophthalmology, Clínica Universidad de Navarra, Pamplona, Spain

## Abstract

Uveitic glaucoma (UG) typically is associated with very high intraocular pressure (IOP) and more intense optic nerve damage than other glaucoma types. This secondary glaucoma requires an early diagnosis and adequate management of both uveitis and glaucoma. It is mandatory to identify the mechanisms of IOP elevation that in many eyes have multiple combined mechanisms. Management of these patients commonly requires an interdisciplinary approach that includes a glaucoma specialist and rheumatologist to control the inflammation and IOP. Glaucoma surgery is required early in these patients due to the high IOP usually present and is less successful than in primary open-angle glaucoma. Recurrent uveitic episodes, multiple mechanism, and the complications associated with uveitis make surgical management of UG challenging. In this review, the management and treatment of UG are updated to clarify the pathogenesis and prevent optic nerve damage.

## 1. Introduction

Patients with uveitis have an increased risk of intraocular pressure (IOP) elevation not only because of the disease but also as a side effect of corticosteroid use [1].

Uveitic glaucoma (UG) includes a range of disorders whose common end result is glaucomatous optic nerve and visual field damage. Compared with primary open-angle glaucoma (POAG), patients with UG are younger and IOP values are higher with acute elevations and varying responses to antiglaucomatous drugs.

The mechanism of UG is complex (different open-angle and closed-angle mechanisms can coexist in the same patient), and management requires careful diagnosis and adequate control of both IOP and inflammation. A multidisciplinary approach is necessary in many cases to achieve a successful outcome.

The very high IOP and the complex interrelation with ocular inflammation explain why many patients with UG require glaucoma filtering surgery sometimes combined with phacoemulsification. Strict control of inflammation increases the chance of success, but surgery for UG historically has been considered refractory because of the increased risk of failure. Otherwise, ciliary body inflammation can result in prolonged postoperative hypotony, making the results of filtering procedures more unpredictable.

This update is intended to assist ophthalmologists who are managing patients with UG. We did not rate the quality of evidence cited but described the study design in many cases.

## 2. Methods

We searched the published peer-reviewed medical literature to identify studies that evaluated UG. Multiple databases were searched, including MEDLINE, EMBASE, Cochrane, ERIC, and EBSCO. The search was limited to articles in English and foreign language publications with English abstracts. Several key words were used including uveitic glaucoma, inflammatory glaucoma, Posner-Schlossman syndrome (PSS), and Fuchs heterochromic cyclitis. All retrieved articles were cross-referenced and citations in the bibliography were retrieved if deemed relevant. Articles displayed in the “related articles” link on PubMed also were used when relevant.

## 3. Physiopathology

The mechanisms that determine an IOP increase in UG are diverse and complex; many are often present simultaneously in the same patient. Open-angle glaucoma (OAG) occurs as a result of mechanical obstruction of the trabecular meshwork by inflammatory cells, proteins, debris, fibrin, or inflammatory precipitates. Additionally, direct inflammation of the trabecular meshwork and/or the effect of corticosteroids on the trabecular meshwork may contribute to the open-angle mechanism of UG [1, 2]. Up to one-third of patients with uveitis treated with corticosteroids may have elevated IOP, and it may be difficult to distinguish between the side effects of the corticosteroids and the underlying inflammation. A family history of glaucoma, rheumatoid arthritis, diabetes, and younger age are considered risk factors of a steroid responder [1].

Secondary angle closure can result from synechial closure, neovascularization of the chamber angle, or seclusion pupillae with subsequent appositional angle closure. Less commonly, angle-closure glaucoma develops when inflammation and edema cause ciliary body forward rotation to close the angle, as in patients with Vogt-Koyanagi-Harada syndrome (VKHS).

These complex interactions cause patients with UG to have high IOP fluctuations and great variability in the therapeutic response.

## 4. Classification

**Figure 1:**
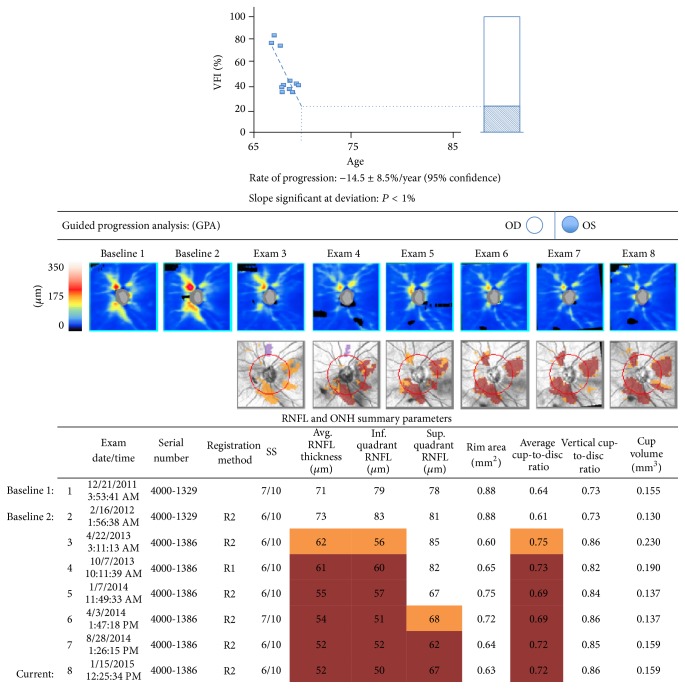
Visual field and OCT progression after recurrent episodes of UG (VFI: Visual Field Index).

Some authors have proposed differentiating between hypertensive uveitis and UG based on the absence or presence of optic nerve damage, but this distinction usually is not applied to secondary glaucomas [3]. Typical hypertensive uveitis, such as in PSS, can cause glaucomatous damage over time in relation to the number, duration, and intensity of the episodes (Figure 1) [4].

## 5. Diagnosis

Recent improvements in the clinical evaluation of the optic nerve and retinal nerve fiber layer (RNFL), such as scanning laser ophthalmoscopy and optical coherence tomography (OCT), and of the angle, such as ultrasound biomicroscopy (UBM) and anterior segment OCT, are as relevant to UG as to other glaucomas.

The higher IOP levels associated with UG may cause apparent structural damage detected by optic disc imaging that disappears when the IOP level returns to normal [3]. Optic disc imaging is a useful way to document the glaucoma status in this type of eye, provided that media opacification does not hamper image acquisition.

However, it is important to consider that uveitis is a major confounding factor in assessing the RNFL thickness. Moore et al. reported substantial RNFL thickening in patients with active uveitis and a thicker RNFL than expected in patients with UG [5], probably related to breakdown in the blood-retinal barriers and increased production of prostaglandin analogues (PGAs). After the inflammation improves, the retinal thickness decreases and thinning of the RNFL and increased cupping can be observed [6]. These changes raise concerns about the comparative value of RNFL scans as a method for detecting and monitoring glaucomatous damage in patients with uveitis.

Normal-appearing measurements of the RNFL thickness in patients with UG should be interpreted cautiously in those with elevated IOP. Physicians should recognize that continued thinning of the RNFL and increased cupping, despite good IOP control in such eyes, might be due to resolution of edema of the RNFL.

Screening for glaucomatous RNFL changes in uveitis must be performed during quiescent periods. Thinning of the inferior quadrant suggests that glaucomatous damage is in fact occurring [7]. Measurement of the RNFL may facilitate detection of signs of damage before disc or visual field changes and therefore identifies a subgroup that should receive more aggressive treatment [7]. In addition, OCT also has become a standard for confirming the diagnosis of macular edema [8].

If the cornea cannot be cleared adequately, UBM and OCT are useful for evaluating the angle [1]. UBM is valuable for evaluating different types of angle-closure glaucoma. This technique currently has an advantage over OCT in that the ciliary body can be visualized as the iridocorneal angle even in the presence of substantial corneal opacification. Visualization of the ciliary body is particularly useful for diagnosing chronic ocular hypotony, which paradoxically may be a late development in patients with chronic UG. It is also very important to evaluate closed-angle glaucoma secondary to anterior rotation of the ciliary body. Anterior-segment OCT also may be helpful to evaluate the length and position of glaucoma drainage device tubes and their relationship with the corneal endothelium [9] as well as to evaluate filtering bleb in eyes that have undergone filtration surgery [10, 11].

## 6. Epidemiology and Etiology

**Figure 2:**
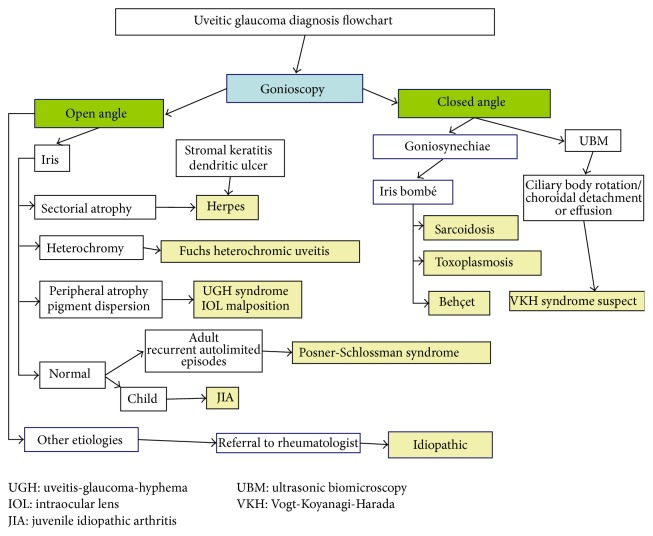
UG diagnosis flowchart.

Glaucoma occurs in around 20% of all patients with chronic uveitis [2]. The incidence and clinical appearance of UG differ according to the disease etiology. The etiology of uveitis varies among different ethnicities and even among regions of the same country [12]. Higher rates are reported in those with rheumatoid arthritis-associated iridocyclitis, Fuchs heterochromic iridocyclitis (27%), sarcoidosis (34%), herpes simplex keratouveitis (54%), zoster uveitis (38%) [2], Lyme-associated uveitis, cancer-associated uveitis [13], juvenile idiopathic arthritis (JIA) (12–35%), Behçet's disease, pars planitis, sympathetic ophthalmia, and syphilis [2]. Acute IOP elevation is also typical in PSS. Some signs are characteristic of specific etiologies and may be helpful to establish a correct diagnosis (Figure 2).

### 6.1. Fuchs Heterochromic Uveitis (FHU)

**Figure 3:**
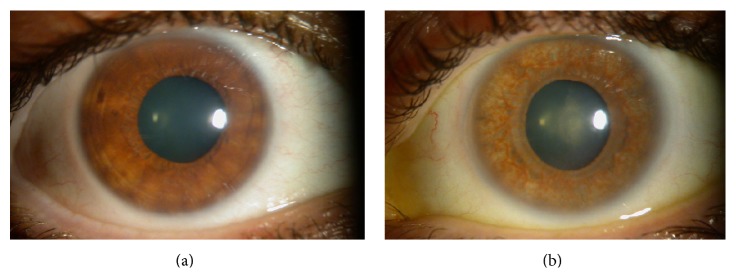
Fuchs heterochromic uveitis in one patient. The right eye is normal. The left hypochromic eye is affected.

FHU was described as the triad of anterior uveitis, heterochromia, and cataract. It is unilateral in 90% of cases and the affected eye is the hypochromic one (Figure 3). The uveitis is chronic and low-grade, without synechiae and with typical small stellate keratic precipitates [1]. Microhyphema after paracentesis, gonioscopy or tonometry (Amsler's sign) is typical of FHU and related to the anomalous vessels in the angle chamber. OAG is present in 13% to 59% of cases. Initially, it can respond to anti-inflammatory and medical treatment, but a filtering surgery is commonly needed to control the IOP. FHU is considered to have a higher risk of failure when associated with UG [14].

Chee and Jap reported that 41.7% of eyes with presumed FHU are cytomegalovirus- (CMV-) positive. Patients with CMV-positive presumed FHU are more likely to be men, be older at diagnosis, and have nodular endothelial lesions [15].

### 6.2. PSS

Glaucomatocyclitic crisis, or PSS, presents typically with unilateral recurrent episodes of mild cyclitis with a few fine keratic precipitates and elevated IOP in the range of 40 to 60 mmHg during episodes that usually resolves spontaneously. The IOP is normal between attacks and the angle is open [15, 16]. The course is commonly benign, but about 25% of patients can develop glaucomatous damage if the number of episodes or the disease duration is sufficiently long (Figure 1) [4].

In two recent studies, more than 50% of aqueous humour samples from eyes with PSS were positive for CMV by polymerase chain reaction (PCR) analysis [15, 17]. Severe endothelial cellular loss and a higher number of eyes requiring glaucoma filtering surgery were observed in patients with CMV-positive PSS [17].

### 6.3. Herpetic Uveitis

**Figure 4:**
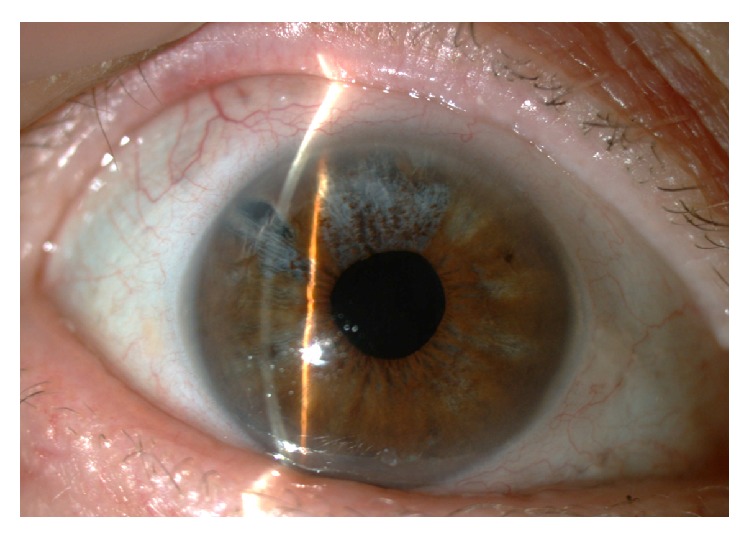
Sectorial iris atrophy is typical of herpetic uveitis.

UG is the most common complication in patients with herpetic uveitis and it is typically unilateral. An acute increase in IOP in the presence of active iridocyclitis is the hallmark of a herpetic etiology, associated with herpes simplex virus or varicella zoster virus. Inflammation of the trabecular meshwork has been proposed as the cause of IOP elevations and is supported by normalization of the IOP after corticosteroid treatment [1]. Diffuse or sectorial iris atrophy is a characteristic of herpetic iritis (Figure 4). The presence of corneal stromal opacities is typical of herpetic stromal keratouveitis and can assist in the etiologic diagnosis. In some severe cases, posterior synechiae and fibrin deposition may be present [1].

### 6.4. JIA

Most patients who develop uveitis have oligoarticular JIA [18]. About one-third of patients with JIA-associated uveitis develop secondary ocular complications such as posterior synechiae, cataract, band keratopathy, glaucoma, or macular edema [18]. The prevalence of glaucoma or ocular hypertension in JIA-associated uveitis has been reported to range from 14% to 42% [1]. Patients with persistent low-grade intraocular inflammation are at the greatest risk for developing glaucoma. OAG and secondary closed-angle glaucoma as a result of formation of posterior synechiae can be present in JIA uveitis. Immunomodulatory therapy such as methotrexate is often necessary to treat the chronic iridocyclitis associated with JIA [18, 19].

### 6.5. VKHS

VKHS typically presents as bilateral panuveitis with dermatologic and central nervous system manifestations. Glaucoma can be present in 18% to 38% of cases. The management of the closed-angle mechanism is especially challenging because edema and anterior rotation of the ciliary body can be present, and these cases do not respond to iridotomy.

### 6.6. Postoperative UG

UG can be present after complicated cataract surgery. Secondary glaucoma can develop because of retained nuclear or cortical lens fragments. Malposition or subluxation of an intraocular lens (IOL) can determine pigment dispersion and elevated IOP. The uveitis-glaucoma-hyphema syndrome is the typical clinical picture and IOL explantation may be required in some cases [20].

## 7. Management

Uveitis is a complex multifactorial ocular inflammatory disease process that often requires a multidisciplinary approach. Successful management requires simultaneous treatment of both uveitis and IOP elevation. Adequate control of inflammation is mandatory and a current mistake is to undertreat the uveitis to avoid the corticosteroid-induced IOP elevation. This conservative approach can result in trabecular meshwork damage secondary to the inflammatory process.

Etiologic treatment may be helpful in some specific etiologies such as herpetic keratouveitis. When present, the angle-closure component must be managed [3].

### 7.1. Anti-Inflammatory Treatment

The first step in UG management is controlling the inflammation, which minimizes the adverse effects of the inflammatory process. In some cases, controlling the uveitis may help reduce the IOP. Patients treated aggressively with anti-inflammatory therapy have a better clinical course of the UG [1].

Corticosteroids are the preferred anti-inflammatory drug used to treat uveitis. It is advisable to start with strong topical corticosteroids such as prednisolone acetate, but periocular or systemic corticosteroids may be required in refractory cases [1, 3]. Rimexolone and loteprednol induce the IOP steroid response less often; however, the anti-inflammatory effect is weaker and in UG it is necessary to use stronger corticosteroids. The chronic inflammation commonly present in FHU does not require continued anti-inflammatory treatment, but it could be useful to use corticosteroids in acute exacerbations of uveitis with transient IOP spikes [21].

Nonsteroidal anti-inflammatory drugs are not usually helpful for treating UG and can partially block the hypotensive effect of some glaucoma medications such as latanoprost and brimonidine [2, 22].

In corticosteroid responders, immunosuppression with drugs such as cyclosporine, azathioprine, methotrexate, or anti-tumor necrosis factor-alpha antibody therapy may be necessary. In these cases, coordination with a uveitis specialist or rheumatologist who is more comfortable in initiating or adjusting systemic immunomodulatory therapy is advised [2].

Cycloplegic agents must be used with anti-inflammatory treatment in some acute uveitic episodes, with the exceptions of PSS and FHU. In case of peripheral anterior synechiae with permanent angle closure, mydriatics and cycloplegics may be contraindicated.

### 7.2. Antiviral Treatment

Antiviral treatment should be prescribed to treat specific etiologies such as herpes simplex or varicella zoster. Topical antiviral therapy is indicated in patients with keratouveitis to prevent viral replication during treatment with topical steroids, but it is considered ineffective in herpetic uveitis. Along with management of glaucoma, long-term antiviral prophylaxis such as oral acyclovir, valacyclovir, or famciclovir usually is required to prevent recurrences. Acyclovir 800 mg twice daily or valacyclovir prophylactically for patients with herpes simplex disease and double the dose for varicella zoster disease have been recommended [1].

Aqueous analysis by PCR recently has been positive for CMV in some patients with PSS and Fuchs heterochromic iridocyclitis [15]. Considering that more than 50% of patients with PSS will be positive for CMV after PCR analysis of the aqueous humor, ganciclovir and valganciclovir have been proposed as etiologic treatments.

Topical ganciclovir effectively clears the viral load, helps control IOP, and preserves the corneal endothelium of patients with CMV-positive PSS. The regimen used was topical 2% ganciclovir solution every 2 to 3 hours daily as induction therapy and every 4 hours for long-term maintenance therapy. All CMV-infected eyes treated with continuous topical 2% ganciclovir had undetectable CMV levels at subsequent analyses. During follow-up, the average number of antiglaucomatous agents decreased but a similar frequency of IOP spikes occurred in both groups.

Patients with CMV-positive eyes with a disease duration exceeding 5 years were likely to require glaucoma surgery. All patients undergoing surgery had CMV-negative PCR results during the IOP attack but had severe peripheral anterior synechiae and pigment clogging [17].

In the same way, 11 patients with PSS with positive CMV PCR analysis of the aqueous humor were treated with 900 mg of valganciclovir twice daily for 3 weeks followed by 450 mg twice daily for a mean period of 20 months. In the first week of treatment, the IOP decreased significantly and remained stable during the entire treatment period. However, two patients had a recurrence after the drug was discontinued. No side effects of therapy developed. Long-term oral therapy with valganciclovir seems to lower the recurrence rate in patients with clinically diagnosed PSS and positive CMV aqueous humor [23].

### 7.3. Antiglaucomatous Drugs

In UG, the effectiveness of antiglaucomatous medical treatment may vary in the presence of inflammation or when combined with mandatory steroid treatment. Less topical medication may be absorbed in the presence of inflammation, and the IOP-lowering effect of most ocular hypotensive agents can vary markedly in uveitis, ranging from no response to profound reductions (70%–80%) with relatively small amounts of ocular hypotensive medication in the occasional uveitic eye with very labile IOP levels [2].

No clinical evidence supports a first-line therapy for UG. Traditionally, topical beta-blockers and CAIs have been considered the first-line agents to treat increased IOP associated with uveitis. PGAs can be used as first-line therapy in UG with controlled uveitis [24, 25]. Systemic CAIs should be considered if topical medications fail to achieve the desired effect [1].

#### 7.3.1. Beta-Blockers

Nonselective topical beta-adrenergic antagonists are considered the first-line agents used to decrease IOP in patients with UG without systemic contraindications [3]. Metipranolol, including the unpreserved preparation, should be avoided because of its association with anterior granulomatous uveitis [3, 26, 27].

#### 7.3.2. PGAs

Controversy exists concerning the use of PGAs in patients with uveitis due to the theoretically higher risk of anterior uveitis, blood-aqueous barrier disruption, cystoid macular edema (CME), and reactivation of herpes simplex keratitis. However, in a comparative study on the efficacy and safety of latanoprost against a fixed combination of brimonidine and timolol in patients with UG, latanoprost was at least as effective as the fixed combination and there were no differences in the rate of inflammatory recurrences and incidence rates of CME between the treatments. The authors concluded that latanoprost is as safe and effective as the fixed combination of brimonidine and timolol for treating UG [24].

A paradoxical reaction after treatment with latanoprost was reported in three patients with UG with increased IOP and recurrent inflammation 7 to 16 days after rechallenging with topical latanoprost. However, all patients had undergone a previous complicated intraocular surgery [28].

Another concern is related to the possible induction of chronic conjunctival inflammation that may have a negative effect in future filtering surgeries. After studying conjunctival cells by impression cytology for inflammatory markers by flow cytometry, Taylor et al. found that the use of topical PGAs does not induce conjunctival inflammation over that already present in patients with UG. This finding supports the use of topical PGAs in patients with UG, indicating that their use is unlikely to adversely affect subsequent glaucoma filtration surgery through induction of chronic conjunctival inflammation [29].

In summary, PGAs and prostamides may be first-line therapy choices in patients with UG, especially in cases of quiescent uveitis without previous complicated intraocular surgery or preexisting CME [1, 24, 25]. In eyes with a history of herpetic keratitis or keratouveitis, PGAs are best avoided [25].

#### 7.3.3. CAIs

The IOP-lowering effect of topical CAIs varies greatly in patients with uveitis [27]. A potential advantage is the possible positive effect in preventing and treating CME coexistent with UG.

Dorzolamide significantly inhibits CAI activity. Irreversible corneal decompensation has been described after topical administration of dorzolamide in patients with underlying corneal endothelial compromise. In patients with preexisting corneal endothelial injury, topical CAIs must be avoided [3].

Acetazolamide is used frequently to manage acute IOP elevations in combination with other antiglaucomatous drugs. It is especially helpful in preparing patients for filtering surgery.

Although an anecdotal case report has reported the additive effect on IOP reduction with the concomitant use of topical and systemic CAIs [30], the general trend is to consider that topical CAIs do not have an additive effect to maximal oral doses of acetazolamide.

#### 7.3.4. Alpha-2 Adrenergic Agonists

Currently, brimonidine, an alpha-2 adrenergic agonist, is considered a useful second-line therapy for patients with glaucoma and it is most often used in combined therapy.

**Figure 5:**
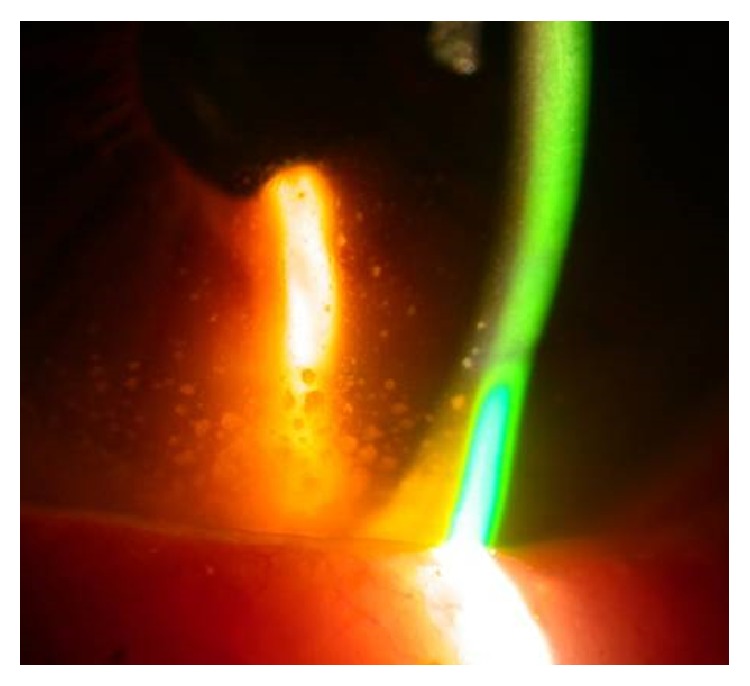
Granulomatous uveitis after long-term apraclonidine treatment.

Granulomatous anterior uveitis has been described after long-term use of apraclonidine and brimonidine. Most cases developed about 1 year after alpha-2 adrenergic treatment and typically an allergic reaction preceded the anterior uveitic episode and the patients had not stopped the treatment after this episode (Figure 5).

Some cases recurred after rechallenging with brimonidine, confirming the causal relationship. Typically, the inflammation resolves rapidly after stopping the alpha-2 adrenergic treatment and with use of topical corticosteroids [31, 32].

When using an alpha-2 adrenergic agonist, it is important to be alert for the first signs of intolerance or allergic reaction and immediately stop treatment. Anterior uveitic reactivation is possible after brimonidine treatment in patients with UG.

#### 7.3.5. Cholinergic Agents

Cholinergic agents or miotics generally are contraindicated for treating UG because of the potential exacerbation of inflammation via blood-aqueous barrier breakdown. In addition, miotics promote development of posterior synechiae, and in patients with synechial angle closure these drugs are generally ineffective given their mechanism of action of increasing trabecular aqueous outflow [27].

### 7.4. Laser Trabeculoplasty

The common angle-closure mechanism in many cases of UG may preclude the use of argon laser trabeculoplasty (ALT). There is also concern about the risk of exacerbating inflammation and trabecular meshwork damage after ALT. ALT currently is not recommended for treating UG [3].

Selective laser trabeculoplasty (SLT) has been suggested as an alternative treatment for UG. Siddique et al. reported a significant IOP reduction after SLT in naïve eyes with UG (19.8% after a 1-year follow-up). SLT was less effective in eyes that underwent a previous glaucoma surgery [1]. However, the complete results and complications have not been published, and currently there is insufficient clinical evidence to recommend SLT to treat UG.

### 7.5. Surgery

If medical management fails to control IOP, surgery is the next step. About 30% of eyes with UG may require surgery [33]. The surgical success rates in UG varies markedly (50%–100%) [13]. There is a consensus that the surgical success rate of filtering surgery is lower for eyes with UG compared with POAG.

As a rule, suppression of inflammation in the perioperative period significantly improves outcomes [12]. Regardless of the surgical modality chosen, all patients require meticulous control of inflammation preoperatively and vigilant monitoring for reactivation postoperatively. Otherwise, the ciliary body can be damaged by inflammation and/or mitomycin C (MMC) use during filtering surgery. Surgeons should exercise caution when recommending irreversible filtering glaucoma procedures and in concomitant use of antimetabolites to avoid prolonged hypotony and the risk of phthisis bulbi.

Preoperatively, although good control of intraocular inflammation for a number of months is ideal, filtration surgery rarely is an elective procedure, and a regimen of preoperative topical or systemic corticosteroid treatment (e.g., 0.5 to 1 mg/kg/day of oral prednisolone) is useful to reduce intraocular inflammation and the inflammatory cells in the conjunctiva [2].

Intraoperatively, antifibrotics during filtering procedures may retard postoperative wound healing. Alternatively, an infraorbital depot of 40 mg of methylprednisolone or intravitreal 4 mg of triamcinolone can be administered at the conclusion of surgery [2].

Postoperatively, a major challenge to successful filtration surgery for uveitis is the accelerated healing that occurs in the presence of postoperative inflammation. However, there is no way to completely eliminate postoperative inflammation and the severity of uveitis may increase postoperatively.

The significant risk factors for surgical failure are male sex, age younger than 45 years, nongranulomatous uveitis and prolonged postoperative inflammation [12].

The choice of the most appropriate surgery depends on patient age, inflammatory activity, previous ocular surgeries, conjunctival scarring, pathophysiology of the IOP elevation, surgeon experience, and postoperative IOP goal.

#### 7.5.1. Trabeculectomy

Classically, trabeculectomy has been the procedure of choice for treating UG, with the exception of aphakic eyes, neovascularization, or poor visual function [3]. Success rates from 50% to 100% have been reported after trabeculectomy to treat UG [13]. Poor success rates with trabeculectomy performed without antiproliferatives have been reported in UG; the standard of care is adjunctive 5-fluorouracil (FU) or MMC in these patients [1]. In UG, Towler et al. reported that after 5 years follow-up, 50% of eyes that underwent trabeculectomy with 5-FU were controlled versus only 30% of eyes in which 5-FU was not applied [34].

Trabeculectomy with MMC is less effective in UG than in POAG. However, Kaburaki et al. did not found differences in the efficacy and safety of trabeculectomy with MMC as the initial ocular surgery in inactive uveitis and POAG, although hypotonic maculopathy was more common in UG [35].

Granulomatous uveitis and previous cataract surgery are considered risk factors for failure after trabeculectomy with MMC [36]. In granulomatous uveitis, fibrotic tissue and granuloma containing Langhans giant cells accumulate in the trabecular meshwork and Schlemm's canal and may obstruct the filtering pathway created by trabeculectomy.

Trabeculectomy with MMC in patients with UG has been associated with a higher risk of cataract progression. Regarding the cataractogenic effect of trabeculectomy [37], chronic ocular inflammation, and continuous corticosteroid treatment also may contribute to more rapid progression of cataract.

The most common complications after trabeculectomy in patients with UG are recurrent inflammation (17.6%) and hypotony (11.8%). Meticulous control of inflammation preoperatively and vigilant monitoring for reactivation is mandatory. Considering the associated damage to the ciliary body in some patients with uveitis, prudent use of MMC is advisable to avoid prolonged postoperative hypotony.

Even though subconjunctival bevacizumab (Avastin, Genentech Inc., South San Francisco, CA) has been used successfully for controlling wound healing after glaucoma filtration surgery, no data have been published on the safety and efficacy of intraoperative use of bevacizumab as adjunct to trabeculectomy in UG [1].

#### 7.5.2. Ex-PRESS Mini-Glaucoma Shunt

The Ex-PRESS glaucoma filtration device (Alcon Laboratories, Fort Worth, Texas, USA) is a metallic implant that provides an artificial channel to drain aqueous into the subconjunctival space. This technology is less invasive than traditional trabeculectomy. The Ex-PRESS shunt does not require a sclerectomy or peripheral iridectomy; hence, there is less inflammation and risk of blockage of the inner window by fibrin, blood, or iris. All of these factors can be advantageous in UG.

In a small preliminary case series of five patients, Lee et al. reported the safety and efficacy of the Ex-PRESS glaucoma filtration device with intraoperative MMC for use in UG. The complete and qualified success rates were 80% and 100%, respectively, after 6-month follow-up. Postoperative hypotony due to ciliary shutdown occurred in 20% of cases, one of which was complicated by choroidal detachment and long-term hypotony maculopathy [38]. Larger trials are warranted to establish the long-term efficacy and safety of the Ex-PRESS for the treating UG.

#### 7.5.3. Nonperforating Deep Sclerectomy (NPDS)

This is an attractive alternative for glaucoma surgery in UG and in steroid-induced IOP elevations with an open angle in that it avoids anterior chamber entry, iris manipulation, and prolonged hypotony. The absence of iris manipulation is of special importance in patients with uveitis and may reduce the risk of postoperative inflammation and hyphema. These complications have been associated with a greater risk of failure after filtering surgery.

The integrity of the trabeculodescemetic window allows controlled outflow of aqueous humor, which reduces the risk of profound and long-term hypotony, and it also has been postulated to prevent egress of cytokines and inflammatory mediators from the anterior chamber into the subconjunctival space, which reduces the risk of inflammation, scarring, and failure of filtering surgery [39–41].

There is increasing evidence that NPDS is probably more appropriate for UG [40–43]. Al Obeidan et al. published the largest prospective study of 33 consecutive eyes with uncontrolled UG treated with NPDS with MMC and implantation of either the T-Flux implant (Ioltech, La Rochelle, France) or SK gel (Corneal Laboratories, Paris, France). After a mean follow-up of 33.2 ± 19.8 months, the IOP decreased from a mean preoperative value of 37.2 mmHg to a mean postoperative value of 14.7 mmHg. Complete success was achieved in 72.7% of eyes and qualified success in 21.2% of eyes. Neodymium (Nd):YAG laser goniopuncture was performed in 36.4% of eyes, after which the iris adhered to the trabeculodescemetic window in one patient. Postoperative complications included cataract progression (27.3%), transient hypotony (18.2%), shallow choroidal effusions (12.1%), and hypotony with persistent maculopathy, hyphema, and decompression retinopathy (3%) [41]. These complications may be more prevalent in patients with UG than in those with POAG.

Regarding trabeculectomy, Dupas et al. showed in a retrospective study that similar midterm control of IOP was obtained by either trabeculectomy with MMC (0.4 mg/mL for 3 minutes) or NPDS with MMC (0.4 mg/mL for 3 minutes) and the T-Flux implant, with similar success rates at 12 months. No significant difference between the results of these procedures was found for postoperative complications or the need for reoperation. However, NPDS required many more postoperative adjustments than trabeculectomy (goniopuncture and needling) and trabeculectomy induced marked, though transient, worsening of intraocular inflammation. Visual acuity scores and postoperative cataract progression requiring phacoemulsification were similar in both groups [42].

Although randomized prospective comparative studies of these two procedures are still necessary, this study suggested that NPDS (with simultaneous use of an implant and MMC) and trabeculectomy with antiproliferative agents are both effective for managing UG. NPDS generates less inflammation during the early postoperative follow-up but requires close monitoring for appropriate adjustment of IOP-lowering interventions, such as goniopuncture or needling. However, trabeculectomy leads to high transient postoperative inflammation but facilitates direct IOP reduction with very few postoperative adjustments and might be indicated in cases in which close monitoring is difficult [42].

**Figure 6:**
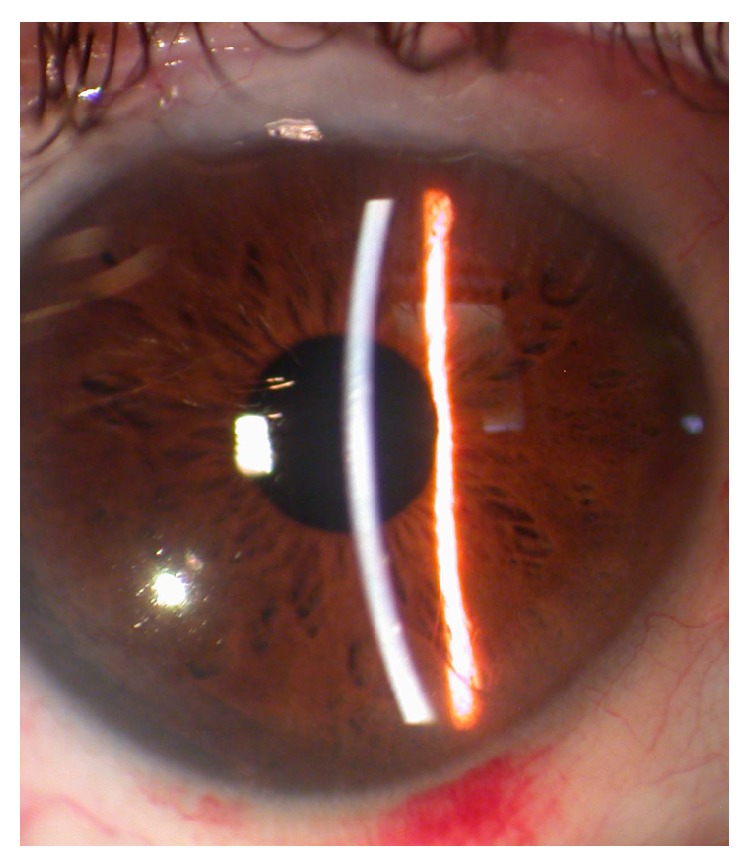
PSS. Two small endothelial precipitates are seen. A conjunctival filtering bleb after NPDS is seen.

More controversial is the indication of filtering surgery in PSS. Campana et al. reported a patient with PSS who underwent NPDS with MMC and the T-Flux implant. Goniopuncture was required 9 months after NPDS. The IOP remained 15 to 16 mmHg without topical treatment and no subsequent episode of ocular inflammation 6 years after Nd:YAG laser goniopuncture [44]. In our personal experience, NPDS in PSS facilitates significant reductions in the number and severity of hypertensive peaks (unpublished data); one patient presented with persistent hypotony maculopathy after NPDS with MMC (Figure 6).

A modified NPDS has been described in JIA. The authors made two circumscribed punctures from Schlemm's channel into the anterior chamber, lateral to the sclerectomy. They used MMC (0.2 mg/mL) on the bare sclera for 1 minute in all cases without an implant and reported that IOP can be reduced sufficiently using standard trabeculectomy with MMC and NPDS with MMC, but trabeculectomy with MMC may be more effective. However, additional surgeries to adjust the IOP were common for both groups. In aphakic children, the modified sclerectomy described earlier appears to be a better technique for avoiding vitreous prolapse [45].

#### 7.5.4. Canaloplasty

This procedure may be of special interest in UG surgery because it acts on an important source of outflow resistance in uveitic eyes exposed to steroids. Glucocorticoids increase IOP via deposition of extracellular matrix material in the juxtacanalicular tissue, leading to thickening of the trabecular meshwork beams, decreased intertrabecular spaces, and a subsequent increase in outflow resistance. Histologic analysis of UG eyes on topical steroids confirmed the trabecular meshwork beam thickening [46]. Canaloplasty expands and maintains a patent Schlemm's canal, increasing the previously reduced intertrabecular spaces.

In a retrospective pilot study of 19 uveitic eyes, canaloplasty with postoperative Nd:YAG goniopuncture was a safe and effective surgery for treating open-angle UG [28]. At the last follow-up visit (mean follow-up time, 2.6 ± 1.1 years), the complete success rate was 73.7% and the failure rate was 15.5%. A 55% reduction in IOP was achieved and the mean number of antiglaucoma drugs decreased from 3.7 ± 0.8 preoperatively to 0.4 ± 1.0 at the last follow-up. The postoperative complications were Prolene suture erosion into the anterior chamber (10.5%), transient hyphema (5.3%), prolonged hypotonous maculopathy after goniopuncture (5.3%), and rapid progression of cataract (5.3%).

The mean number of steroid drops was 0.5 ± 0.6 in the preoperative period and 0.7 ± 1.4 6 months postoperatively.

Canaloplasty is a promising technique for UG, because it expands the intertrabecular spaces, targeting an important source of outflow resistance in uveitic eyes exposed to glucocorticoids [33].

#### 7.5.5. Glaucoma Drainage Devices (GDD)

These devices often are considered the first choice for UG surgery, especially in etiologies such as JIA [47]. In patients with extensive peripheral anterior synechiae, the tube should be placed in the sulcus rather in the anterior chamber to avoid endothelial trauma.

**Figure 7:**
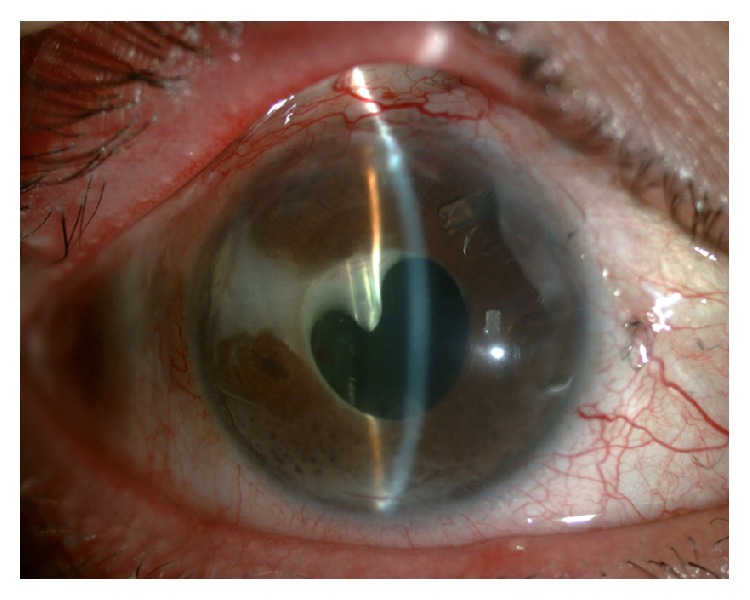
Ahmed valve tube occlusion by fibrin exudation in a patient with UG.

Owing to its unidirectional valve mechanism, implantation of Ahmed glaucoma valve (AGV) (AGV; New World Medical Inc., Rancho Cucamonga, CA, USA) may be more convenient because of the lower risk of immediate postoperative hypotony. Success rates of 77% and 50% have been reported with AGV in UG after 1 and 4 years of follow-up, respectively [48]. Encapsulated bleb (43%), transient hypotony (43%), and hyphema (21%) are the short-term complications most commonly described after AGV implantation in UG [48, 49]. Occlusion of the tube by inflammatory materials and corneal decompensation also has been associated with AGV implantation in eyes with UG (Figure 7) [48].

Preoperative use of corticosteroids may improve the surgical success of the AGV in UG. Mata et al. suggested prescribing 1 mg/kg/day prednisone preoperatively until the inflammation is controlled. In the postoperative period, oral corticosteroids are taped tapered over 4 weeks [50].

Nonvalved GDDs such as the Baerveldt implant (Abbott Laboratories Inc., Abbott Park, IL, USA) have been recommended. The cumulative probability of success was greater with a Baerveldt GDD than after trabeculectomy for UG. There was a significantly higher frequency of early complications in the trabeculectomy group compared with the GDD group; however, no significant differences were seen in the frequency of late postoperative complications between groups. The most common postoperative complications after implantation of a Baerveldt GDD were hypotony and CME, but there were no differences between the Baerveldt implant and trabeculectomy. The authors concluded that implantation of the Baerveldt GDD was more likely to maintain IOP control and avoid reoperation for glaucoma compared with trabeculectomy with antifibrotic therapy in eyes with chronic inflammatory glaucoma [13].

The Molteno aqueous shunt (Molteno Ophthalmic Ltd., Dunedin, New Zealand) also has been recommended for primary surgical treatment in UG. Vuori reported a qualified success rate of 85% after 4-year follow-up. The IOP decreased continuously during the first year postoperatively, and the medication was slowly tapered even up to 3 years postoperatively. Therefore, the author suggested postponing further surgical interventions during the first postoperative year after Molteno implantation in UG, even if the IOP is not controlled. Persistent hypotony was present in 6.66% of cases and corneal decompensation in 3.33% of cases [51].

In summary, GDDs are one of the preferred first-line surgeries in UG. The AGV, Molteno, and Baerveldt GDDs have been used with good success rates, but no studies have compared if nonvalved or valved GDDs are preferable in UG.

#### 7.5.6. iStent

In a mixed series of secondary glaucoma cases including four cases with steroid-induced glaucoma, Buchacra et al. reported that the Glaukos iStent (Glaukos iStent, Glaukos Corporation, Laguna Hills, CA) is a safe and effective surgical option for secondary OAG, but they did not present isolated data for steroid glaucoma [52]. Good results were achieved with Glaukos trabecular bypass in one case of IOP elevation induced by steroid treatment after laser in situ keratomileusis [53].

After preliminary studies, the Glaukos iStent may be an attractive alternative for steroid-induced glaucoma, considering the microinvasive and reversibility characteristics of the procedure, although larger well-designed studies are needed to confirm this conclusion.

#### 7.5.7. Trabectome Surgery

Shimizu et al. performed trabectome surgery in a subgroup of patients with UG and reported a success rate of 75%, but more details about the safety and efficacy of trabectome in UG were unavailable [12].

#### 7.5.8. Goniotomy

The procedure has been suggested for refractory glaucoma associated with chronic childhood uveitis [54–56]. The largest series included 54 goniotomies in 40 eyes, with the predominant diagnosis of juvenile rheumatoid arthritis (mean age at surgery, 10.3 years). Overall surgical success was achieved in 72% of cases (complete success, 55%). Phakic eyes, fewer peripheral anterior synechiae, age younger than 10 years, and eyes with no previous surgery had significantly better outcomes. The most common postoperative complication was mild and transient hyphema (80%) [55].

Goniosurgery is low risk and effective for refractory glaucoma complicating chronic childhood uveitis. For some authors, it should be considered the surgical procedure of choice for this condition, although almost half of patients will need glaucoma treatment postoperatively. The surgical outcome is affected adversely by increased age, peripheral anterior synechiae, previous surgeries, and aphakia [55]. However, goniotomy requires considerable skill and experience and is best avoided by specialists who do not perform it regularly [3].

Randomized comparative studies are needed to determine the efficacy and safety of goniotomy compared with trabeculectomy or GDD surgery.

#### 7.5.9. Cyclophotocoagulation

Cycloablative techniques can be used to decrease aqueous production by destruction of the ciliary body using transscleral or intraocular diode or Nd:YAG laser cyclophotocoagulation. Unfortunately, cycloablative procedures can exacerbate inflammation and lead to postoperative hypotony and phthisis bulbi. The rate of hypotony after cyclodiode laser in uveitis (19%) is higher than in any other secondary glaucomas [2].

Schlote et al. reported a series of 22 patients who underwent transscleral diode laser cyclophotocoagulation (TDLC) for UG or scleritis-associated glaucoma. The IOP was controlled in 77.3% of eyes, although 63.6% of cases needed more than one treatment with TDLC. The investigators did not observe reactivation of inflammation, persistent hypotony, or phthisis bulbi in any case [57]. Good results also were reported in a case of UG secondary to JIA treated with TDLC [58]. Although preliminary studies have reported encouraging results with TDLC in UG, it should be the last resort for refractory glaucoma in eyes with poor visual potential in which conventional drainage surgery has failed or is impossible because of the ocular anatomic characteristics.

#### 7.5.10. Cataract and UG Surgery

Cataract is very common in patients with uveitis. The optimal sequence of surgery with concomitant cataract and UG is controversial. Cataract surgery can compromise the success of trabeculectomy [59], but combined glaucoma and cataract surgery increases the risk of postoperative inflammation and may be less successful than isolated filtering procedures [3, 14].

If combined glaucoma and cataract surgery is indicated, good control of the inflammation is mandatory preoperatively and postoperatively. The use of antimetabolites at the time of combined surgery reduces the proliferative response [60]. A meticulous and minimally invasive surgical procedure also can help increase the surgical success, but the evidence is insufficient to recommend a specific filtering surgery for combined procedures in this kind of patient.

It is also essential to be alert for detecting and treating postoperative complications such as hypotony, athalamia, and choroidal detachment. Stronger and longer postoperative steroid treatment usually is required. Intensification of anti-inflammatory treatment may be necessary in case of recurrent uveitis.

#### 7.5.11. Iridotomy

**Figure 8:**
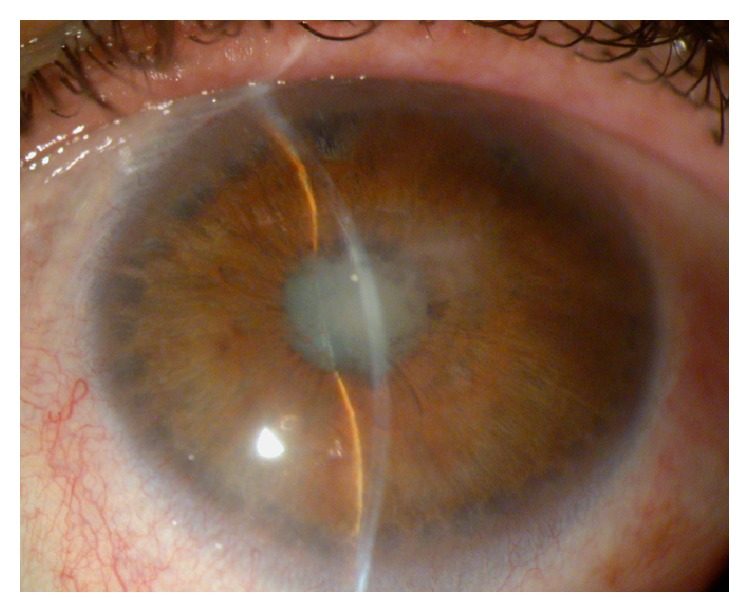
Iris bombé and pupillary seclusion in a patient with UG. Peripheral iris burns after argon laser iridoplasty are seen.

**Figure 9:**
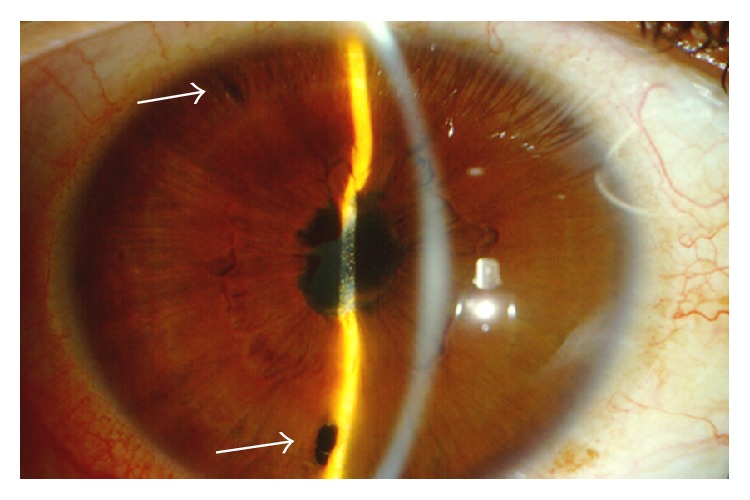
Iris bombé after two Nd:YAG laser iridotomies (arrows).

Nd:YAG laser peripheral iridotomies are indicated in cases of iris bombé and angle closure secondary to posterior synechiae (Figure 8). In UG, Nd:YAG laser iridotomy has an increased incidence of failure (61% in some retrospective studies) [61]. Spencer et al. reported that the median survival of Nd:YAG peripheral iridotomy was 85 days, with most failures occurring within the first 20 days. Those investigators recommended multiple (at least two) or large iridotomies (Figure 9), aggressive treatment with topical steroids and cycloplegics, and close monitoring of patients with frequent early review. If the iridotomy closes, there should be early consideration for a surgical peripheral iridectomy [61].

Recurrent herpetic keratouveitis has been described after argon laser iridotomy [62] and after Nd:YAG laser peripheral iridotomy [63]. The causal relationship is difficult to establish because patients were being treated with topical corticosteroids and in one case with latanoprost, previously related to recurrent keratouveitis. Both cases resolved with oral acyclovir and discontinuation of latanoprost. Preventive treatment with oral acyclovir has been suggested if iridotomy is required in patients with UG associated with herpes virus.

Most studies that have reported the outcomes of trabeculectomy or GDD in uveitic eyes are not specific to uveitis-associated angle closure, and this warrants further investigation in more targeted studies. As in primary angle-closure glaucoma, phacoemulsification combined with goniosynechialysis may be an alternative in patients with uveitis with closed-angle glaucoma, although it is expected to be less successful when chronic peripheral anterior synechiae are present [47].

Other causes of secondary closed-angle glaucoma such as anterior ciliary body rotation, annular ciliary body detachment, or uveal effusion require specific surgical approaches.
